# Interpreting the dominant signature of inhomogeneous mixing resulting from dry-air entrainment in clouds

**DOI:** 10.1126/sciadv.aeb6751

**Published:** 2026-05-20

**Authors:** Nithin Allwayin, Gregory Roberts, Elise Rosky, Kenny Bala, Aaron Bansemer, Keyvan Ranjbar, Raymond A. Shaw

**Affiliations:** ^1^ Department of Physics, Michigan Technological University, Houghton, MI 49931, USA.; ^2^ Scripps Institution of Oceanography, University of California San Diego, La Jolla, CA 92037, USA.; ^3^ National Research Council Canada, Ottawa 10587, Canada.; ^4^ NSF National Center for Atmospheric Research, Boulder, CO 80307, USA.

## Abstract

How cloud droplets evaporate when mixed with the dry surrounding air is fundamental to cloud optical properties and lifetime. We find from observations in cumulus clouds made during the ESCAPE field campaign that this mixing process appears strongly inhomogeneous-like, where a subset of droplets evaporate completely as mixing proceeds, rather than all droplets partially evaporating. We visualize the microphysical properties in a two-dimensional evaporation-phase-relaxation space and find that a diffusive turbulent-evaporation model is able to capture the dynamic evolution of the entrainment process. The results indicate that the first evaporating droplets humidify the region around the cloud so that the unmixed dry air rarely reaches the core, explaining why most mixing events appear inhomogeneous. A mixing slope parameter also confirms the nature of the mixing process. On the basis of the inhomogeneous mixing model, we propose a simple parameterization of cloud optical properties suitable for coarse-resolution models.

## Teaser

Cloud response to entrainment visualized in a 2D evaporation-phase-relaxation space supports a simple model for mixing.

## INTRODUCTION

Rising thermals in a cloud draw in dry air from the surroundings, leading to dissipation of its liquid water content (*1*–*4*). How this process proceeds determines how long clouds persist and affects precipitation, development, and the reflection of sunlight (*5*–*9*). Two extreme limits of this mixing process are defined: inhomogeneous mixing, where a fraction of the droplets completely evaporates, and homogeneous mixing, where all droplets partially evaporate (*10*, *11*). Historically, most climate models have used entrainment parameterizations that assume the mixing to be instantaneous and therefore homogeneous (*12*, *13*). However, recent observations indicate that inhomogeneous-type mixing is prevalent, and it remains largely unaccounted for in most parameterization schemes (*14*–*17*). Recently, attempts have been made to incorporate assumptions of inhomogeneous mixing, and more substantial research has been dedicated to quantifying and parameterizing the intermediate scenario between homogeneous and inhomogeneous mixing (*18*, *19*), but considerable gaps remain. Consequently, the mixing process and its effect on cloud microphysics contribute to a large uncertainty in climate models (*20*, *21*). Fully understanding the mixing process is nontrivial, with studies identifying cases of homogeneous, inhomogeneous, and intermediate mixing scenarios (*19*, *22*–*28*). However, what is important is the cloud microphysical response to the entrainment, particularly how mixing influences the mean radius and the cloud droplet number concentration.

Using observational cloud data and an idealized mixing model, we investigate the cloud response to entrainment by treating mixing as a dynamic, time-evolving process. We use cloud microphysical data from the Cloud Droplet Probe (CDP-2) (*29*) and the Holographic Detector for Clouds (HOLODEC) (*30*) collected during the Experiment of Sea Breeze Convection, Aerosols, Precipitation, and Environment (ESCAPE) field campaign in Houston, TX (*31*). The CDP-2 measures all cloud droplets in the range 2 to 50 μm in 1-s intervals, corresponding to an ~100-m spatial average (*32*), whereas HOLODEC instantaneously measures 6 to 2000 μm droplets in an ≈10 cm^3^ localized, centimeter-scale sample volume (*33*). HOLODEC is ideally suited to study the mixing problem (*25*). However, during the deployment, HOLODEC faced operational issues, and so the data from the instrument was sparse across the field campaign. For these reasons, we primarily use the cloud droplet size and number concentration data from the CDP-2 using HOLODEC wherever available to verify that the CDP-2 results are not biased due to spatial averaging.

Entrainment studies have often used mixing diagrams that plot the relationship between mean droplet diameter and number concentrations. The Damköhler number representing the ratio between the turbulent mixing and droplet response timescales is also commonly used to differentiate the mixing types (*11*, *24*, *34*, *35*). Lehmann *et al.* (*22*) introduced a transition length scale, where mixing changes from inhomogeneous to homogeneous; and they recognized that both the single-droplet evaporation time and the phase relaxation time are relevant to the mixing problem. Theoretical analysis using an idealized diffusion-evaporation model by Korolev *et al.* (*36*) and Pinsky and Khain (*37*) further demonstrated how mixing diagrams show an incomplete picture, which cannot always distinguish the mixing types. They proposed a two dimensional phase space analysis to understand mixing. Fries *et al.* (*38*) reached a similar conclusion using a statistical evaporation-mixing model, comparing it to direct numerical simulations and HOLODEC field observations. We find that the two-dimensional parameter space as defined in these papers (*38*, *39*) is ideal to investigate the entrainment mixing process. To provide additional insight, we also analyze the data in terms of the mixing slope developed by Yeom *et al.* (*28*). An idealized model that includes evaporation and mixing via turbulent diffusion, adapted to the ESCAPE cloud conditions, is able to capture the essential microphysical signatures identified with these analysis methods. This suggests an interpretation for the dominance of inhomogeneous-like mixing, and provides a basis for straightforward parameterization of cloud microphysical response to mixing. The term ‘inhomogeneous-like’ is used intentionally, as we have found that the cloud microphysical response is similar irrespective of the mixing scenario; from a microphysical perspective, inhomogeneous mixing dominates.

## RESULTS

On the basis of the analysis of ESCAPE data from HOLODEC and CDP-2, we find the microphysical response to mixing predominantly inhomogeneous-like, with the droplet radius remaining nearly constant. This conclusion is based on the mixing diagram analysis for each of the cloud passes and evaluating the CDP-2 cloudy points in a phase space consisting of dimensionless parameters describing the phase-relaxation time and the timescale for single-droplet evaporation (theory and results discussed in the “Mixing diagrams,” “Theory,” and “$|R|-{\mathrm{Da}}_{p}$” phase space sections, respectively). To understand this behavior, we developed a simplified turbulent-diffusion-evaporation model that simulates the mean characteristics of the turbulent mixing process (“Turbulent-diffusion-evaporation model” section). Mixing initially is purely inhomogeneous, leading to the complete evaporation of droplets at the cloud edges. This process moistens the cloud edges, effectively shielding the cloud from further dry environmental air intrusion. Mixing then mostly proceeds as a series of dilution events, which leads to an interpretation of inhomogeneous mixing in the cloud core. The few homogeneous mixing events that occur are with nearly saturated environmental air, making their microphysical response indistinguishable from inhomogeneous mixing. Last, a mixing-slope analysis validates the results, demonstrating how such a simplified model can capture the key entrainment features (“Mixing slope analysis” section). In Discussion (“Parameterizing entrainment mixing” section), we propose an entrainment parameterization based on the observed cloud microphysical response.

### Mixing diagrams

**Fig. 1.**
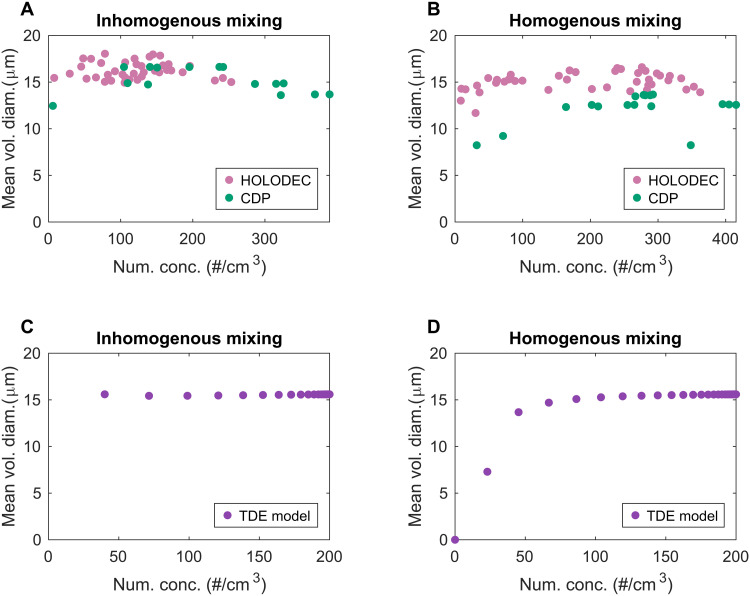
Mixing diagrams plot the relationship between cloud droplet count and mean droplet diameter for all points along the cloud breadth. Here we present examples of the different mixing scenarios observed. Subplot (**A**) is illustrative of most of the cloud transects, and strongly inhomogeneous and subplot (**B**) shows an example of the rare instances where there is some homogeneous-like mixing signature. Subplots (**C**) and (**D**) show how similar mixing behaviors emerge in the turbulent-diffusion-evaporation model.

Mixing diagrams plot the relationship between cloud droplet number concentration and the mean volume diameter of the droplets. For most cloud transects analyzed, the mean volume diameter remains relatively constant as cloud points become diluted toward the cloud edge, suggesting that inhomogeneous mixing dominates. We observe a slight reduction in mean volume diameter at the most diluted regions of the cloud, a dip that comes with inhomogeneous-type mixing events (*40*). In rare cases, this reduction is more pronounced, indicating a small shift toward slight homogeneous mixing. Figure 1 (A and B) illustrate this behavior, with the left plot representing most of the cloud cases with a strong inhomogeneous signature, and the right plot demonstrates an example of the few cases where there is a hint of homogeneous mixing. Notably, we also observe that, where both CDP-2 and HOLODEC measurements exist, there is consistency in the type of mixing identified. In other words, the CDP-2 measurements are not strongly biased toward inhomogeneous mixing signatures due to spatial averaging over cloudy filaments (*35*). This builds our confidence that we can reliably use CDP-2 data from the full set of flights for the analysis.

### Theory

A more comprehensive view of the mixing process can be obtained by considering it within the two-dimensional phase space suggested in various theoretical and computational investigations (*38*, *39*, *41*). We adopt the terminology of Pinsky *et al.* (*39*), which defines the potential evaporation parameter, *R*, relating to the single droplet evaporation time, and the phase relaxation Damköhler number, ${\text{Da}}_{p}$ (*39*). For a mixing event between the cloud and the environment (denoted by subscripts c and e), *R* is defined as

$$R=\frac{S_{e}}{A_{2}q_{\text{lc}}}$$ (1)

where $S_{e}$ is the saturation deficit in the environment and $q_{\text{lc}}$ is the liquid-water mixing ratio of the cloud. The phase relaxation Damköhler number, ${\text{Da}}_{p}$ is given by

$${\text{Da}}_{p}=\frac{\tau_{\text{mix}}}{\tau_{p}}$$ (2)

Here, $\tau_{\text{mix}}$ is the characteristic turbulence mixing timescale and $\tau_{p}$ is the supersaturation relaxation timescale. The phase relaxation time for a cloudy volume is defined as

$$\tau_{p}=\frac{1}{4\pi \left(\rho_{w}/\rho_{a}\right)A_{2}A_{3}n_{d}r_{m}}$$ (3)

where $n_{d}$ is the droplet number concentration, $r_{m}$ is the mean volume radius, $\rho_{w}$ is the density of water, and $\rho_{a}$ is the density of air. $A_{2}$ and $A_{3}$ are thermodynamic coefficients, exhibit minimal variation, and can therefore be assumed to remain constant. $A_{2}$ is nondimensional, and $A_{3}$ has dimensions of $m^{2}$ $s^{-1}$. The mixing time can be approximated using

$$\tau_{\text{mix}}={\left[\frac{l^{2}}{\varepsilon }\right]}^{1/3}$$ (4)

where *l* is the length scale of mixing and ε is the eddy dissipation rate estimated from the root mean square of the vertical component of velocity in the cloud segment, $w_{\text{rms}}$ (see the Supplementary Materials for details).

The potential evaporation parameter can be interpreted as the ratio of the phase relaxation time of the droplets to the single droplet evaporation time, $\tau_{d}$ given by (*38*)

$$\tau_{d}=\frac{r_{m}^{2}}{2A_{3}|S_{e}|}$$ (5)

With this insight in mind, we can interpret the space as representing the relative roles of turbulent mixing ($\tau_{\text{mix}}$), single-droplet evaporation ($\tau_{d}$), and supersaturation adjustment (i.e., phase relaxation, $\tau_{p}$). Earlier cloud studies suggested that the smaller of the two microphysical timescales dominate the response to mixing (*22*) but did not consider the behavior in a two-dimensional phase space. Here, we analyze an extensive set of in situ cloud measurements from multiple flights through cumulus clouds within this theoretical framework of Pinsky *et al.* (*39*) and Fries *et al.* (*38*).

### $|R|-{\mathrm{Da}}_{p}$ phase space

We estimate the potential evaporation parameter *R* and the Damköhler number ${\text{Da}}_{p}$ for all CDP-2 cloud samples from the 395 cloud passes across 13 research flights. The distribution of points in this two-dimensional space reveal their mixing characteristics. In this theoretical framework, a cutoff value of *R* can be specified, above which all droplets completely evaporate during the mixing process and leave the mixed volume unsaturated. For a mixing fraction, χ of 0.5, this region is ∣R∣>1. Below this threshold, 0<∣R∣<1, the cloud liquid water is only partly evaporated and diluted by entrainment. This mixing can be homogeneous, inhomogeneous, or intermediate, depending on the mixing and cloud response timescales. Figure 16 in (*39*) and figure 4 in (*38*) nicely illustrate this concept. We use the absolute value of *R* [different from (*39*)] for intuitive visualization. As the cloud mixing fraction, χ, increases, the threshold of ∣R∣ above which the cloud completely evaporates exceeds *1*. This threshold is given by the relation

$$|R_{c}|=\frac{\chi }{1-\chi }$$ (6)

For example, when the mixing volume is 90% cloud, the $|R_{c}|$ is 9.

**Fig. 2.**
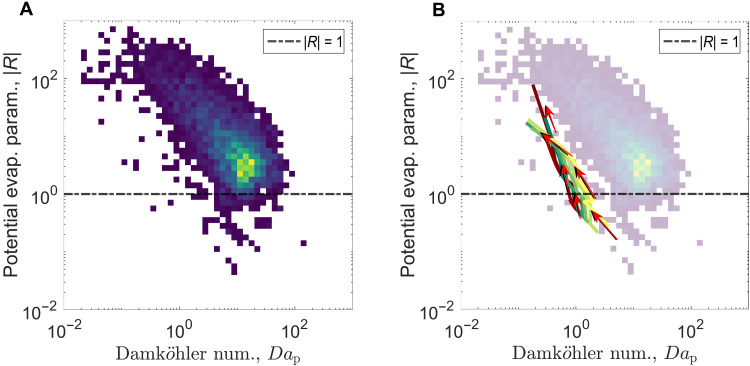
$|R|-{\mathrm{Da}}_{p}$ phase space for comprehensive analysis of the mixing process. Subplot (**A**) is a two-dimensional histogram of all cloud samples from 395 cloud traverses across 13 research flights, measured with the CDP-2 instrument. (**B**) Evolution of mixing in the dimensionless space for different cloud points in the domain of the turbulent-diffusion-evaporation model. The red arrows give the general direction of evolution for the cloud points.

For each point in a cloud traverse, we calculate ∣R∣ and ${\text{Da}}_{p}$. The distribution of all the cloudy volumes is represented as a two-dimensional histogram (Fig. 2A). If mixed with an equal volume of environmental air, all points with ∣R∣>1 should evaporate completely. Nearly all of the sampled cloud exist in this limit, implying a mixing fraction, χ>0.5. Deeper in the cloud, a higher mixing cloud fraction is plausible. However, as we move closer to the cloud edge, this large χ assumption likely does not hold. What explains this seeming paradox that clouds occupy a region of the $|R|-{\text{Da}}_{p}$ phase space that suggests they should rapidly dissipate, and yet they somehow evolve in a way that allows them to persist for times much larger than the mixing time (e.g., large eddy time)? To better understand this behavior in the phase space, we develop a simple model of entrainment mixing, treating the average effects of turbulence as a diffusive mixing process.

### Turbulent-diffusion-evaporation model

Entrainment occurs as turbulent eddies mix the cloud with its surrounding environment. This mixing proceeds simultaneously across multiple length scales—from the engulfing eddy to the Kolmogorov scale. However, the average effects of this turbulent mixing across a can be reasonably approximated as diffusive [e.g., refer to section 2.8 (*42*)]. To model entrainment, we consider a cloud transect at a constant altitude and simulate entrainment mixing on both sides as a diffusive process. Since mixing in this setup is symmetric, it is only required to analyze half the segment. To align with the field measurements, where the clouds are at least 1-km thick, the model domain is set to 1 km, equally divided between the cloud and the environment, with grid points spaced 10 m apart. A 10-m mixing length scale is also used for the observational dataset, as the penetration depths of the entrained parcels are observed to be along this length scale (*40*).

**Fig. 3.**
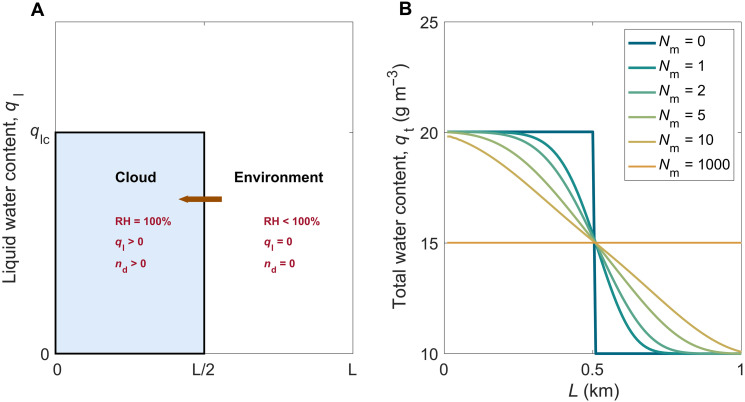
Turbulent-diffusion-evaporation model captures the average behavior of the turbulent mixing process. The subplot (**A**) shows the schematic at the time, *t* = 0 for the mixing model. The left and right regions represent the cloud and the environment. In each step of the mixing process, the adjacent grid boxes mix, and subplot (**B**) shows the evolution of the total water mixing ratio with successive mixing. The evolution of the total water mixing ratio is diffusive and reaches an equilibrium value after about 1000 mixing cycles.

Figure 3A shows the model setup. Here $q_{l}$ and $n_{d}$ are the liquid water content and number concentration of cloud droplets. The model treats turbulent mixing as a diffusive process as illustrated by Fig. 3B. The total water starts as a step function, but with the number of mixing events $N_{\text{mix}}$, the fields are spread out by the turbulent eddies. This is analogous to specifying a turbulent-diffusion coefficient. We achieve this in the model by implementing discrete mixing steps. In each step of the mixing process, adjacent grid boxes are mixed. Initially, this only affects the grid points at the boundary between the cloud and the environment, but as the process continues, more grid points are influenced. When the mixing reaches the domain edge points, we assume one mixing cycle to be complete. Figure 3B shows how the total water content becomes more uniform as the number of mixing cycles increases.

During entrainment the total water mixing ratio ($q_{t}$) and the liquid water potential temperature ($\theta_{L}$) are conserved. Their conservation equations are solved to estimate how much liquid water, $q_{\text{lm}}$, remains in each grid volume after each mixing step. Three scenarios arise: complete evaporation ($q_{\text{lm}}\leq 0$), partial evaporation ($q_{\text{lm}}>0$), and no evaporation. What is relevant are the latter two, with partial evaporation being the result of either of the mixing scenarios—homogeneous or inhomogeneous. To model this, we use a simple criterion: If the mixing timescale between the two grid boxes is greater than the thermodynamic cloud response time, then droplets are affected before the mixing completes, resulting in inhomogeneous mixing. Conversely, if the mixing time is shorter, then droplets evaporate uniformly, representing homogeneous mixing. Specifically, if $\tau_{\text{mix}}>\text{max}\left(\tau_{p},\tau_{d}\right)$, then the mixing is inhomogeneous, and if $\tau_{\text{mix}}\leq \text{max}\left(\tau_{p},\tau_{d}\right)$, then the mixing is homogeneous. When there is no evaporation, the droplets are just redistributed among the grid cells and show up as inhomogeneous in the mixing diagram.

This model, although highly idealized, allows us to explore the evolution of microphysical properties representative of the ensemble-mean behavior of the cloud edge. The model is inspired by that of Pinsky *et al.* (*39*), although the details of its implementation are somewhat different. The objective here is to confront the model with measurements, thereby allowing us to determine whether it can provide insight into observed cloud behavior. The cloud droplet diameters in the model are assumed to be gamma-distributed as a starting point, so all cloud grid boxes are initialized with this distribution. The droplet number concentration and mean volume diameter, turbulent dissipation rate, water vapor mixing ratio, relative humidity of the environment, temperature, and pressure are the inputs to the model. The mean values of these quantities from the ESCAPE data are used to initialize the model. Further details can be found in the Supplementary Materials.

How would a mixing diagram evolve in the model? We generate mixing diagrams (treating each grid volume as cloud points) at each mixing time step. Consistent with the field observations, they look almost always inhomogeneous, as illustrated in Fig. 1C. Occasionally, we find a few time steps where the mixing becomes slightly homogeneous as illustrated in Fig. 1D. Thus, the simple model appears to effectively capture the key microphysical responses observed for real clouds.

We now use the model to better understand how entrainment evolves. Initially, all droplets at the cloud edge evaporate completely, humidifying the near-cloud-edge region. As mixing progresses, this region gets close to saturation, starting to act like a barrier to the dry environmental air. In other words, subsequent mixing between grid volumes is influenced by the moistening from the previous events, drawing parallels to increasing cloud mixing fraction in the “Theory” section. This effect becomes more pronounced as we go deeper into the cloud. When fully saturated, there is no more droplet evaporation and droplets are simply redistributed (i.e., diluted) between the two grid volumes. In other words, a humid shell forms as mixing proceeds, and then most mixing events are dilution events that show up as inhomogeneous-type mixing in a mixing diagram. Note that as the near-cloud regions begin to saturate, the cloud response time increases as well. Mixing events of both types occur here, but the microphysical response is not distinguishable due to the high relative humidity of the mixing air. One might say that the concepts of homogeneous and inhomogeneous mixing lose their meaning when a cloud mixes with a humid, preconditioned environment.

In the phase space, the cloud edge volumes from the model have values of ∣R∣>1, indicating their complete evaporation during the initial mixing stage (Fig. 2B). The $|R_{c}|$ threshold is 1 for the model as grid volumes mix equally. As mixing proceeds and the relative humidity (RH) of the surrounding air increases, ∣R∣ starts to correspondingly decrease. The phase response time increases due to this moistening as well, making the points move down and to the left in this space. Once a point goes below ∣R∣=1, there is no complete evaporation during mixing, and we can start to trace the evolution in the phase space. In this limit, the mixing can be both homogeneous and inhomogeneous. ∣R∣ now increases as a result of the decreasing liquid water, and this continues until all the droplets evaporate, shifting the cloud edge. We analyzed the model with different input configurations to understand how sensitive these results are to the input conditions (additional details are provided in the Supplementary Materials). A similar trend is observed in all cases, the location of points shifts in the space but, importantly, the slope of the cloud evolution remains largely consistent. Note that the mixing rate and so the entrainment rate is assumed constant here.

### Mixing slope analysis

We validate our results using the mixing slope parameter, $\gamma_{s}$, defined by Yeom *et al.* (*28*) as

$$\gamma_{s}=\frac{{\text{log}}_{10}\tau_{p}}{{\text{log}}_{10}q_{l}}$$ (7)

**Fig. 4.**
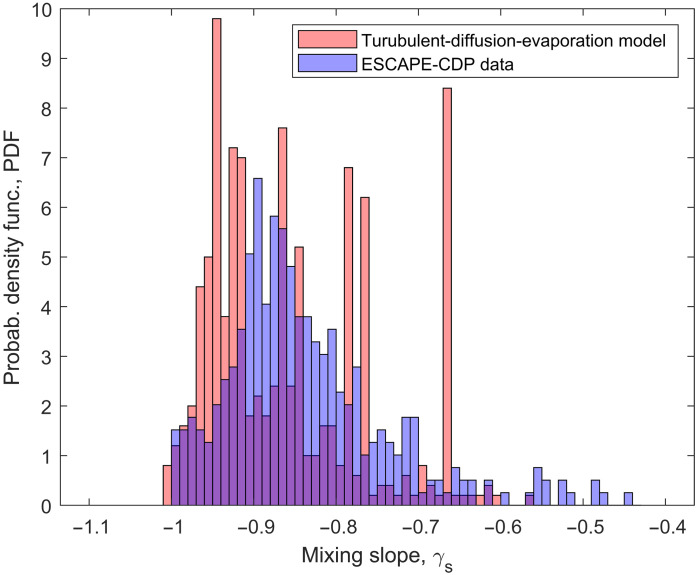
The mixing slope parameter specifies values for the limits of inhomogeneous and homogeneous mixing. This plot shows PDFs of slope parameters from the entrainment mixing model and from cloud droplet measurements made using the CDP-2 during the ESCAPE field campaign. Both distributions peak near a value of −0.9, which is close to the inhomogeneous limit of −1, and have a skewed tail signifying the few events where mixing shifts toward the homogeneous limit of −1/3.

This slope parameter has values of −1 and −1/3 for extreme inhomogeneous and homogeneous mixing events, respectively. The mixing slope parameters for all the mixing steps in the model are calculated and compared to the values derived from the ensemble of cloud transects from the CDP-2 data. Figure 4 illustrates the probability density function (PDF) of the slope values for both the model and CDP-2 data. The mode of the PDF for the CDP-2 data is around −0.9, indicating a strong tendency toward inhomogeneous mixing. The PDF is positively skewed, reflecting a few homogeneous-like mixing events during the process. However, this should be treated with caution, as some clouds sampled in Houston were not isolated cells and were part of a larger system. It is possible that the size distributions could represent a group of clouds with different microphysical properties, which could show up as homogeneous-like mixing signatures (*43*, *44*). In addition, a few anomalous points in the CDP-2 dataset could also bias the mixing slope to homogeneous mixing, as we have just above 10 points for most cloud transects (see the Supplementary Materials for more details).

The model is highly idealized, yet its PDF exhibits a qualitative similarity to the real data, with most mixing scenarios being predominantly inhomogeneous, peaking around −1 and showing a positive skewness that signifies occasional shifts toward homogeneous mixing. The fraction of these homogeneous-like mixing events slightly increases in the model with reduced droplet number concentration and larger mean volume radius and with a higher turbulent dissipation rate. The width of the droplet size distribution appears to have little influence on this behavior (more details are provided in the Supplementary Materials).

## DISCUSSION

### Dominance of inhomogeneous-like mixing in entrainment processes

Mixing diagram analysis reveals that the cloud response to dry air entrainment is strongly inhomogeneous-like in microphysical measurements made during the ESCAPE field campaign. To get a complete picture of entrainment, we analyze these measurements in the two-dimensional phase space based on the single-droplet evaporation time and the phase relaxation Damköhler number. Most cloud volumes are found in a regime, where direct mixing with the environment in equal proportions would rapidly dissipate the cloud. This suggests that these volumes have undergone mixing events, where the cloud fraction consistently exceeds that of the environmental air. It is consistent with the idea that the entrained air becomes moistened as mixing progresses, thereby diminishing the influence of the environmental air with each mixing step. This leads to the formation of a humid shell around the cloud, helping to resist its dissipation. The microphysical response is strongly inhomogeneous-like with the mean diameter remaining relatively constant. The phrase inhomogeneous-like is used because even homogeneous mixing with a humid environment leads to a nearly indistinguishable microphysical outcome.

To investigate this further, we developed a simple, idealized turbulent-diffusion-evaporation model that treats the average effects of turbulence as diffusive to simulate the mixing process. The model is initialized with the observed average values of cloud droplet mean volume diameter, number concentrations, and turbulence properties. The mixing diagrams from the model align with those from the real clouds. We tracked the evolution of cloud points in the phase space and find it to have a slope similar to the observed cloud data. The model confirms the formation of a humid shell, acting to reduce the environmental air contribution in the later mixing steps. Once a humid shell forms, mixing mostly proceeds as a series of dilution events, with no considerable change in the mean diameter. We validated these results using a mixing slope parameter and confirm for the field and model data, the inhomogeneous nature of the mixing process. The fact that such a rudimentary turbulent-diffusion model, which neglects details of the dynamics of mixing and instead only captures the evolution of the ensemble-mean properties of the cloud edge, is able to match an extensive set of observed cloud properties is remarkable. It suggests that diffusive mixing occurring over a finite time, as noted for example by Su *et al.* (*45*), are the essential factors needed to describe the microphysical response to entrainment.

The microphysical response to entrainment is largely governed by the relative humidity of the entrained air. In our model, this dry air evaporates all droplets, saturating the volume. Mixing this nearly saturated air results in dilution events indistinguishable from inhomogeneous mixing. Representing this mixing process using a mean RH value can show up as homogeneous mixing, as it bypasses the intermediate steps. Similarly, averaging across distinct droplet populations may also produce signatures resembling homogeneous mixing (*43*, *44*). This could be a reason for the homogeneous mixing signatures reported in previous studies (*18*). The analysis is independent of the vertical variation of the entrainment rates and its effect on the cloud liquid water. The model starts with an assumed cloud liquid water content at a given altitude. However, this liquid water content can be subadiabatic as a result of prior mixing events from lower altitudes or from cloud top entrainment. The magnitude of this deviation from the adiabatic value depends on the number and the entrainment rate of the prior mixing events. We start with an assumed liquid water content (which could be taken to be a subadiabatic cloud core if preferred) and simulate the lateral mixing process at the cloud edges as is observed in the field (*3*, *46*). The turbulence-diffusion-evaporation model can be extended to include the vertical circulation and cloud-top entrainment effects to represent subadiabatic core conditions. Regardless, in all cases, whether or not there have been prior mixing events, the fingerprint of entrainment on the microphysics remains inhomogeneous in nature.

In summary, the mixing starts purely inhomogeneous and a subset of all droplets evaporate. As mixing progresses and the cloud boundaries are moistened, mixing can be homogeneous or inhomogeneous; however, because the entrained air has high humidity, there is little microphysical distinction between the two types. Once near saturation, mixing mostly proceeds as dilution events that redistribute the cloud droplets. The microphysical response is inhomogeneous-like, with nearly constant droplet diameter and varying degrees of dilution of cloud volumes, consistent with previous studies (*39*, *40*, *47*).

### Parameterizing entrainment mixing

There is growing observational evidence that this inhomogeneous-like microphysical response is prevalent for radiatively important clouds such as stratocumulus and cumulus and across a variety of climatically important zones including Western Atlantic Ocean, Mediterranean Sea, Indian Ocean, and Southern Ocean (*14*, *20*, *21*, *24*, *25*, *28*, *35*, *40*, *43*, *47*–*52*). The radiative impacts on a cloud-by-cloud basis are relatively large, with the difference in cloud radiative forcing between the adiabatic and subadiabatic conditions as high as $100\;W\;m^{-2}$ (*21*). The scenarios where a homogeneous mixing response in stratocumulus clouds is observed to become stronger with increasing distance below the cloud top are likely a result of large-scale variations in cloud-top entrainment rate (*24*, *28*, *53*). This interpretation is strengthened by recent holographic measurements of local droplet size distributions (*43*) and is consistent with controlled laboratory measurements that confirm the dominance of inhomogeneous mixing and how it can appear as homogeneous-like in nature when averaged over different conditions (*44*). In (*44*), it has been pointed out that the microphysical response to mixing can appear “globally homogeneous,” but that is the result of averaging over very large length scales, such as in stratocumulus clouds with substantial variations in entrainment rate at cloud top. As shown in that work and the results presented here, the actual mixing process is inhomogeneous or at least inhomogeneous-like. Similarly, studies have reported that in cumulus clouds, homogeneous mixing increases with altitude; however, the microphysical response is inhomogeneous-like (*16*, *54*). In light of these findings and the theoretical analysis presented here and by others (*37*, *38*, *55*) that provides an interpretation for the dominance of an inhomogeneous-like microphysical signature, we suggest that it should be the default representation of entrainment mixing in coarse-resolution models.

The ubiquity of inhomogeneous-like mixing suggests a relatively straightforward method for parameterizing the microphysical response to entrainment and mixing. Conceptually, it first uses the model’s prediction of the amount of entrainment to calculate the reduction in liquid water content, e.g., using eq. S7. Then, the change in droplet concentration resulting from that change is calculated by assuming constant droplet diameter. The new droplet concentration and the constant droplet diameter, in turn, can be implemented in a calculation of optical depth. Quantitatively, because the droplet diameter is large compared to the wavelength of visible light, the optical depth at a given cloud height is given approximately by $\tau_{h}=\pi n_{d}d^{2}/2$. The liquid water content is $q_{l}={\pi \rho }_{l}n_{d}d^{3}/6$, so if we eliminate $n_{d}$, then we obtain

$$\tau_{h}=\frac{3q_{l}}{\rho_{l}d}$$ (8)

This equation allows a change in $\tau_{h}$ to be calculated directly from a change in $q_{l}$. Stated another way, the dominance of inhomogeneous-like mixing, for which $q_{l}=q_{l}\left(n_{d}\right)$ with constant *d*, implies that the liquid water content susceptibility is $S_{\tau }=\partial \text{ln}\tau /\partial \text{ln}q_{l}=1$, stronger than the value of 2/3 for homogeneous mixing (*25*, *55*).

To represent a cloud of thickness (*h*), we can integrate the Eq. 8 along the vertical extent of the cloud ($\tau =\int_{0}^{h}\tau_{h}\mathrm{dz}$). By adapting the derivation of cloud optical profiles from (*56*), we can rewrite the optical thickness of the cloud (τ) as a function of the reduction of liquid water path (LWP) owing to entrainment ($f_{\text{ad}}={\text{LWP}}_{\text{obs}}/{\text{LWP}}_{\text{ad}}$), cloud droplet number concentrations ($n_{d}$) and cloud thickness (*h*) (for details of the derivation, see the Supplementary Materials): $\tau \propto f_{\text{ad}}^{2/3}n_{d}^{1/3}h^{5/3}$.

When inhomogeneous mixing occurs, not only is there a change in liquid water content, represented by $f_{\text{ad}}$, but there is also a reduction in the droplet number concentration that is equal to $f_{\text{ad}}n_{d,\text{ad}}$, where $n_{d,\text{ad}}$ is the adiabatic (undiluted) droplet concentration. Therefore, the total response of τ is proportional to $f_{\text{ad}}$, which is consistent with the $S_{\tau }=\partial \text{ln}\tau /\partial \text{ln}q_{l}=1$ given above.

### Concluding remarks

We observed inhomogeneous-type mixing predominantly occurring in cumulus clouds based on cloud microphysical data from CDP-2 and HOLODEC data collected during the ESCAPE field campaign. To interpret these observations, we used the two-dimensional phase space of (*39*) and (*38*) and implemented a simple diffusive turbulence-evaporation model. Our findings suggest that entrainment is influenced by prior moistening near cloud boundaries, leading to the emergence of inhomogeneous-like mixing as the main mixing mechanism. This study reinforces previous investigations highlighting how inhomogeneous-like mixing dominates. The mounting evidence support the use of this framework as the basis for entrainment parameterizations, particularly for representing cloud optical properties in coarse-resolution models.

## MATERIALS AND METHODS

The data used in this paper was collected during the Experiment of Sea Breeze Convection, Aerosols, Precipitation, and Environment (ESCAPE) field campaign in Houston, TX. Cloud measurements were taken by instruments aboard the Convair NRC Convair-580 from the National Research Council (NRC) Canada. The cloud droplet size distributions for the mixing diagram analysis were from the CDP-2 and HOLODEC. The CDP-2 measures all cloud droplets in the range 2 to 50 μm in 1-s intervals (*32*), whereas HOLODEC instantaneously measures 6 to 2000 μm droplets in ≈10 cm^3^ sample volume (*33*). The HOLODEC instrument had water contamination on the windows due to heating issues, and the high droplet concentrations degraded the beam quality, pushing many droplets close to the detection threshold. In addition, the laser performance degraded over time due to the extreme temperatures encountered in Houston, further affecting the data quality. For these reasons, we primarily use the cloud droplet size and number concentration data from the CDP-2 using HOLODEC to supplement the analysis wherever available.

To estimate ∣R∣, ${\text{Da}}_{p}$ and other variables, the static air pressure was measured using a Honeywell PPT2 Pressure Transducer and the static air temperature with a Rosemount 102 Total Air Temperature Probe. Wind data were collected via the AIMMS20 Airdata Probe. Two Licor instruments (Licor 840a and Licor 7000) gave the water vapor mixing ratio, and a Vigilant Dew Point Chilled Mirror Hygrometer gave the dew point measurements to estimate the relative humidity of air (*57*).

Cloud transects were taken at constant altitudes for these cumulus-congestus cloud systems. We focus on clouds spanning widths of 1 km or more and limit our analysis to the warm cloud regime. With the airplane traveling at ~100 m s^−1^, each cloud transect includes at least 10 data points (1-Hz sampling). The segments were automatically selected using a continuous liquid water content threshold of 0.0005 g m^−3^ for at least 10 s. In total, there are 395 segments from 13 research flights.

## Supplementary Materials

### This PDF file includes:

Supplementary Text Figs. S1 to S4 Tables S1 to S3 References
