# Supplementary material for: Interpreting the dominant signature of inhomogeneous mixing resulting from dry-air entrainment in clouds
Source: Sci Adv. 2026 May 20;12(21):eaeb6751. doi: 10.1126/sciadv.aeb6751 (PMC13189094; doi:10.1126/sciadv.aeb6751)
Supplement: Supplementary file 1 — Supplementary Text Figs. S1 to S4 Tables S1 to S3 References [file sciadv.aeb6751_sm.pdf]

Science Advances
AAAS

Supplementary Materials for

Interpreting the dominant signature of inhomogeneous mixing resulting from dry-air entrainment in clouds

Nithin Allwayin *et al.*

Corresponding author: Nithin Allwayin, nallwayi@mtu.edu; Raymond A. Shaw, rashaw@mtu.edu

Sci. Adv. **12**, eaeb6751 (2026)
DOI: 10.1126/sciadv.aeb6751

This PDF file includes:

Supplementary Text
Figs. S1 to S4
Tables S1 to S3
References

Supplementary Text

Conservation equations for the Turbulent-Diffusion-Evaporation Model

The variables conserved in the mixing process are the total water mixing ratio (q_t) and the liquid water potential temperature (θ_L). These conservation equations are solved to obtain the liquid water mixing ratio (q_{lm}), water vapor mixing ratio (q_{vm}) , and temperature (T_m) of the two volumes after they mix. For mixing between two grid points 1 and 2, the conservation equations are,

$$q_{tm} = \chi_1 q_{t1} + (1 - \chi_1) q_{t2} \tag{S1}$$

and

$$\theta_{Lm} = \chi_1 \theta_{L1} + (1 - \chi_1) \theta_{L2}, \tag{S2}$$

where χ_1 is the fractional contribution from grid box 1 to the mixed volume. The total water mixing ratio can be decomposed to the liquid and vapor components respectively,

$$q_{lm} + q_{vm} = \chi_1 \left[q_{l1} + q_{v1} \right] + (1 - \chi_1) \left[q_{l2} + q_{v2} \right]. \tag{S3}$$

Similarly, at a constant altitude, the liquid water potential temperature is approximately, $\theta_L \approx T - L_v/C_p q_l$, where T is the temperature. Consequently,

$$T_m - \frac{L_v}{C_p} q_{lm} = \chi_1 \left[T_1 - \frac{L_v}{C_p} q_{l1} \right] + (1 - \chi_1) \left[T_2 - \frac{L_v}{C_p} q_{l2} \right]. \tag{S4}$$

The set of equations can be closed by the Clausius-Clapeyron equation,

$$e_v = 611.3 \exp \left[\frac{L_v}{R_v} \left(\frac{1}{273.15} - \frac{1}{T} \right) \right], \tag{S5}$$

and

$$q_v = 0.622 \left(\frac{e_v}{P - e_v} \right). \tag{S6}$$

Here e_v and q_v represent the water vapor pressure and mixing ratio at temperature T and pressure P. L_v is the latent heat of vaporization of water, C_p is the specific heat capacity of dry air, and R_v is the gas constant for water vapor.

If we approximate a linearized Clausius-Clapeyron equation, we can solve these equations analytically to get an expression for the liquid water mixing ratio after the mixing process, given by

$$q_{lm} \approx \chi_1 q_{l1} - (1 - \chi_1) \frac{q_{v1} - q_{v2} - c_2 (T_1 - T_2)}{1 + L_v c_2 / C_p}, \tag{S7}$$

where c_2 is a constant that depends on the initial thermodynamic conditions. More details on this derivation are given by Yang et al. (*58*) and Shaw et al. (*59*)).

More information on the cloud transects from ESCAPE.

The cloud transects analyzed in this study are identified automatically by segmenting clouds with widths of 1 km or more, using a threshold liquid water content of 0.0005 g m^{-3}. To ensure that the focus remains on warm cloud conditions, we selected only segments below an altitude of 4 km, where the 0 °C isotherm was generally observed during the campaign. Additional details for reproducing these results are provided in Tables S1 and S2.

More information for calculating the potential evaporation parameter, *R*

To calculate the potential evaporation parameter, R for the CDP data, the environment saturation deficit, S_e is estimated from the relative humidity values estimated from the Vigilant Dew Point Chilled Mirror Hygrometer. The value is derived from the mode relative humidity value calculated for 15 points outside the cloud segment under consideration. The liquid water content, q_l is derived from the CDP data.

Input values to the Turbulent-Diffusion-Evaporation model, *R*

The primary inputs for the model include the initial cloud droplet number concentration, n_d, mean droplet diameter, $\bar{d}$, and the energy dissipation rate, ε. Model input values are selected based on average values observed in CDP-2 measurements. Figure S1 displays histograms of the observed values for liquid water content,q_l, r.m.s. of vertical velocity, w_{rms}, number concentration,n_d, and mean volume diameter, $\bar{d}$. The eddy dissipation rate can be determined by the large eddy turnover time and scales as:

$$\varepsilon \approx \frac{w_{\mathrm{rms}}^3}{L} \tag{S8}$$

where L is the characteristic length scale and w_{rms} is the r.m.s. of the vertical velocity. Using $w_{\mathrm{rms}} = 2$m/s for $L = 1$km yields $\varepsilon = 0.008$ m^2s^{-3}. For n_d and $\bar{d}$, we use values of 200 cm^{-3} and

15 μm, respectively, resulting in a liquid water content q_l of 0.3970 g m^{-3}. Detailed input values are provided in Table S3.

Sensitivity of turbulent-diffusion-evaporation model to input parameters

To ensure robustness of our analysis, we perform sensitivity tests to look at the response of the turbulent-diffusion-evaporation model to the input parameters. Since the input variables are interdependent, we vary them in pairs, keeping the rest fixed, and evaluate the effect on the mixing slope parameter, γ_s. The results are shown in Figure S4. Mixing is strongly inhomogeneous for all cases, with the least negative slope parameter close to -0.9, and the fraction of homogeneous-like mixing events shows modest sensitivity. It increases slightly with reduced droplet number concentration and larger mean volume radius (Figure S4(a)), and with higher turbulent dissipation rate (Figure S4(b)). A similar trend is observed for smaller initial relative humidities (Figure S4(c)), while the droplet size distribution width (shape parameter) does not appear to impact the mixing behavior (Figure S4(d)).

Goodness of fit for the mixing slope parameter, γ_s

The mixing slope parameter, γ_s is determined using the ensemble of cloud transects from the CDP-2 data. We select segments 1 km wide and above, and this means at times the number of data points available to perform the linear fit is close to 10, and just satisfies the lowest threshold required for good linear fitting. To ensure our results are not affected by the small sample size we look at the distribution of coefficient of determination (R^2), slope uncertainty ($SE(\gamma_s)$) and residuals for the entire sample (Figure S2). The tests indicate high confidence in the determined slopes with over 81 percent of the segments having a coefficient of determination score greater than 0.9. The slope uncertainty, a measure of the standard error of the estimated slope, is around 0.08 or an 8% error. Further, the residual values are small and are evenly distributed around 0. We also find that the cases where the fit quality are lower are the ones that show slight deviation to homogeneous mixing, as described in the manuscript.

$|R| - Da_p$ phase space- Cloud core vs Cloud edge

To complement the analysis of all cloudy points across 13 research flights from CDP-2 shown in the manuscript, we divide each cloud segment into core and edge regions using an automatic threshold of 70 percent of maximum liquid water content in each segment. Examining the distribution of core versus edge points in the two-dimensional $|R| - Da_p$ phase space reveals distinct regional characteristics (Figure S3). Nearly all core points fall within $|R|$ values between 1 and 10, indicating mixing ratios greater than 0.5. The majority of points in the cloud edge region exhibit a similar trend but occupy a broader region in the phase space with larger $|R|$ and smaller Da values.

The diffusive-mixing model reproduces this behavior: with more mixing, the saturation ratio of the entrained air increases with corresponding decrease in liquid water content of the cloud, resulting in higher $|R|$ values. At the same time, the reduced number concentration increases the phase relaxation time thereby increasing the Damköhler number,Da.

Derivation of cloud optical thickness, τ

The cloud optical thickness for a cloud of height h is given by

$$\tau = \int_0^h \frac{3q_l}{\rho_l d} dz, \tag{S9}$$

where the liquid water content q_l is defined as

$$q_l = \frac{4}{3}\pi r_m^3 n_d. \tag{S10}$$

Expressing the droplet diameter $d = 2r_m$ in terms of q_l from Equation (S10), we substitute into Equation (S9) to get

$$\tau = \int_0^h \frac{3q_l}{\rho_l \times 2\left[\frac{3q_l}{4\pi n_d}\right]^{1/3}} dz. \tag{S11}$$

Simplifying, we obtain

$$\tau = \int_0^h \frac{3}{2\rho_l}\left(\frac{4\pi n_d}{3}\right)^{1/3} q_l^{2/3} dz. \tag{S12}$$

Assuming a relatively constant entrainment rate, the liquid water content increases linearly with height, scaled by the sub-adiabatic fraction f_{ad}:

$$q_l = f_{\text{ad}}\Gamma_{\text{ad}}z. \tag{S13}$$

Here, $\Gamma_{\text{ad}}(T, p)$ represents the adiabatic rate of increase of liquid water content with height. Substituting this into the expression for τ, we have

$$\tau = \int_0^h \frac{3}{2\rho_l}\left(\frac{4\pi n_d}{3}\right)^{1/3} (f_{\text{ad}}\Gamma_{\text{ad}}z)^{2/3}\, dz. \tag{S14}$$

Assuming all terms except height are constant for a constant entrainment rate, the integral simplifies to

$$\tau = A f_{\text{ad}}^{2/3} n_d^{1/3} h^{5/3}, \tag{S15}$$

where the constant A is defined as

$$A = \frac{3}{2\rho_l}\left(\frac{4\pi}{3}\right)^{1/3} \Gamma_{\text{ad}}^{2/3}. \tag{S16}$$

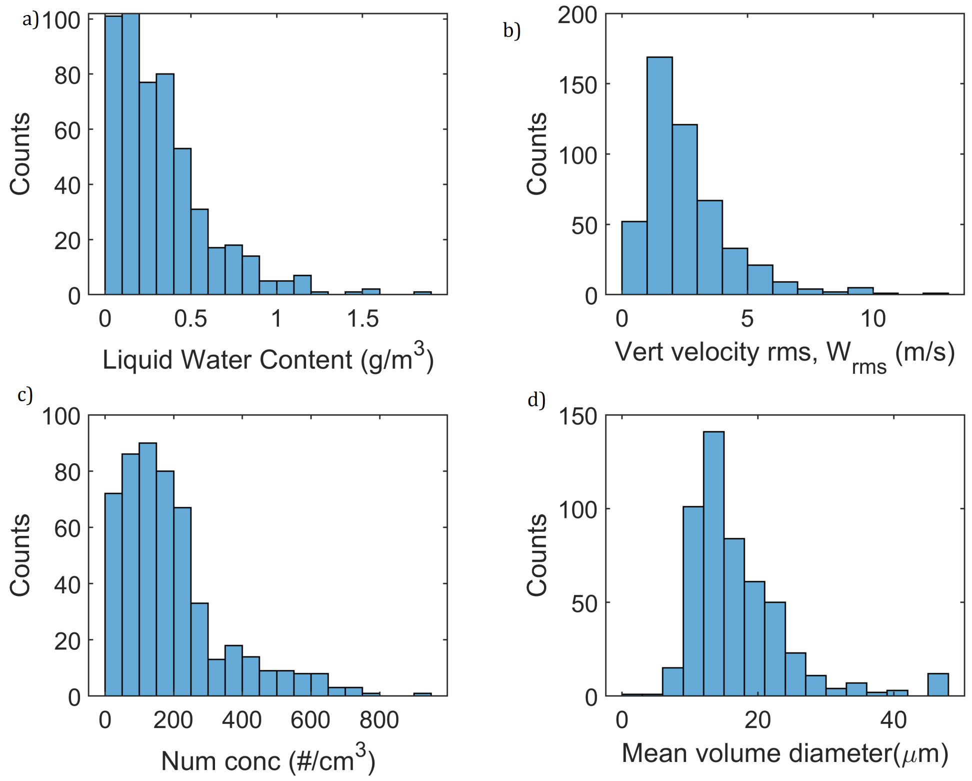

Figure S1: **Histograms of observed properties for all cloud points from selected CDP-2 segments during the ESCAPE field campaign.** (a) Liquid water content (q_l), (b) root-mean-square vertical velocity (w_{rms}), (c) droplet number concentration (n_d), and (d) mean volume diameter ($\bar{d}$)

.

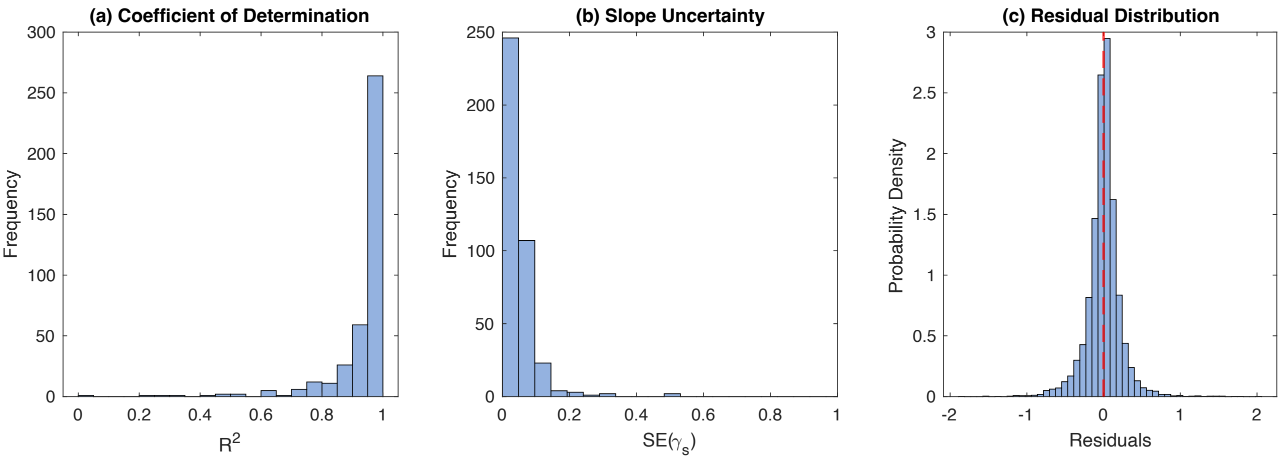

Figure S2: **Goodness of fit parameters for the mixing slope parameter,** γ_s (a) coefficient of determination, (b) slope uncertainty, and (c) residual distributions from the ensemble of CDP-2 cloud segments.

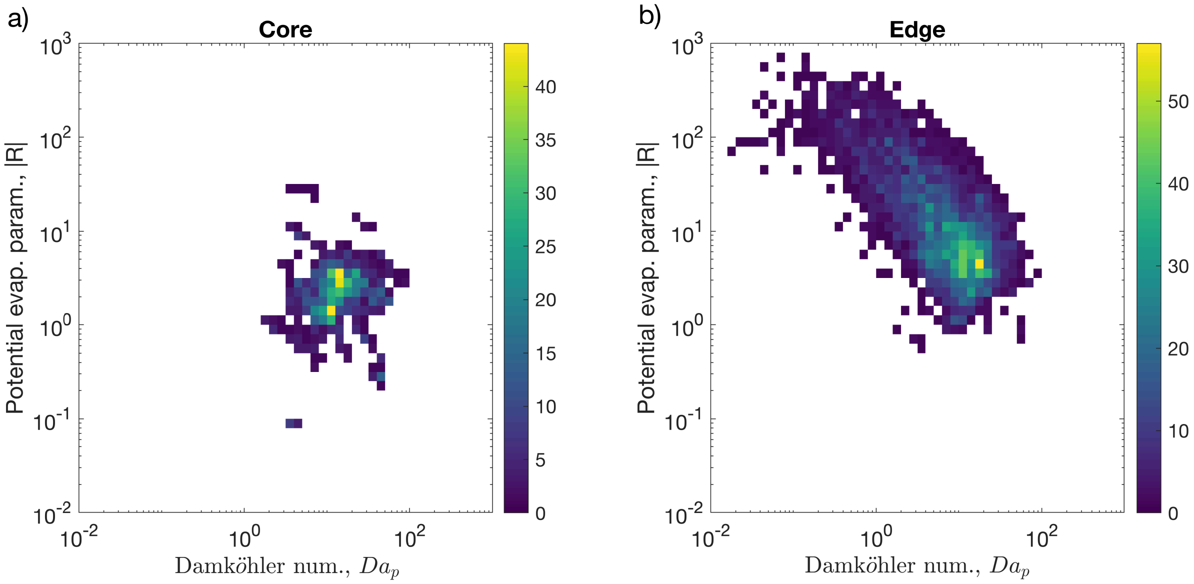

Figure S3: $|R| - Da_p$ **phase space comparing cloud core vs edge characteristics for the CDP-2 data.** Subplot (a) is a 2-D histogram of all cloud samples across the core of the cloud and (b) is the the same for all the cloud samples at the edge.

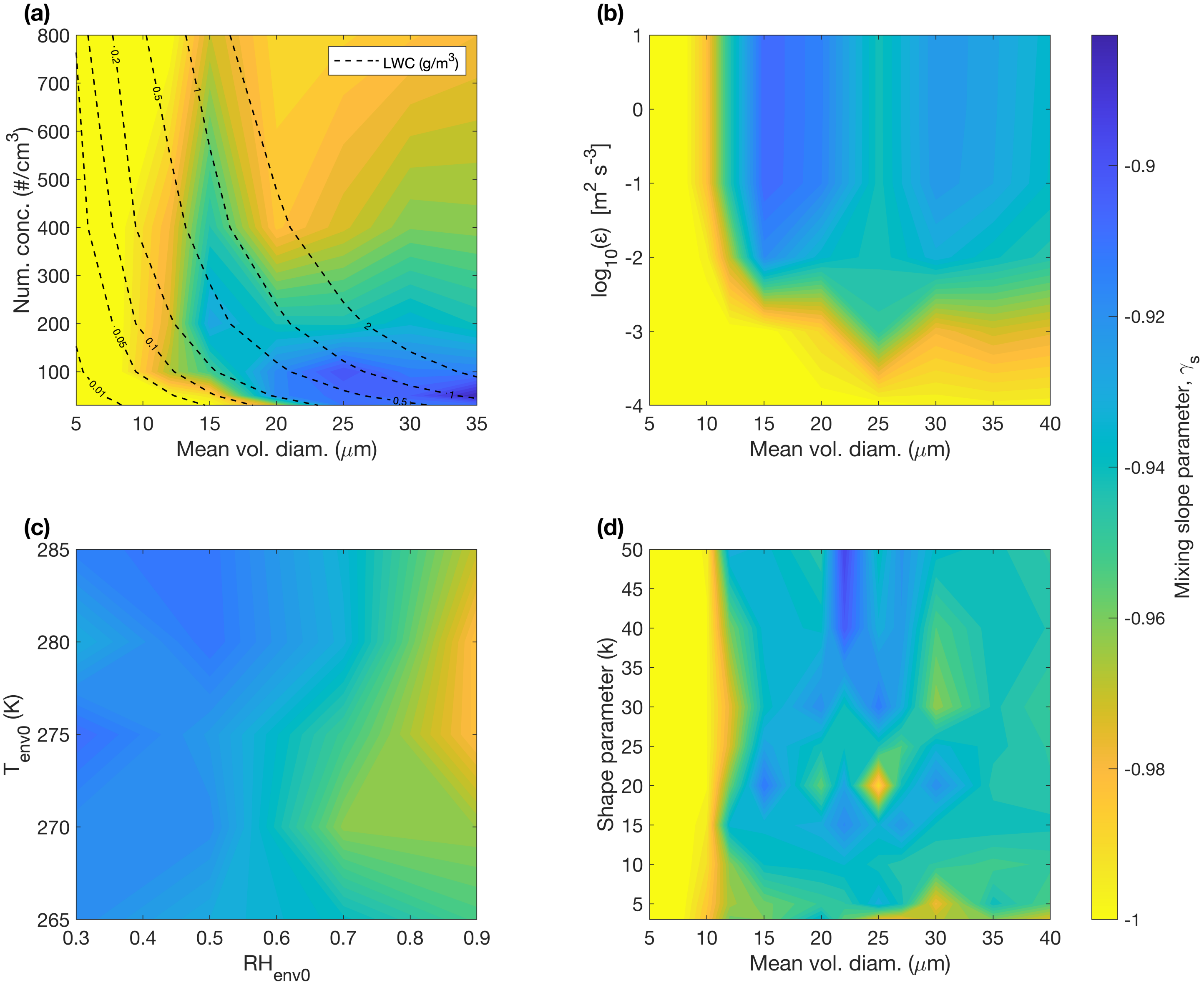

Figure S4: **Sensitivity of turbulent-diffusion-evaporation model to different input parameters, quantified by the mean mixing slope parameter, $\langle\gamma_s\rangle$, across four two-dimensional parameter spaces.** (a) droplet number concentration versus mean volume diameter, with dashed contours indicating constant liquid water content, (b) eddy dissipation rate versus mean volume diameter, (c) initial environmental temperature versus initial relative humidity, and (d) mean volume diameter versus gamma shape parameter. All other parameters are held at baseline values.

Flight no.	No. of seg.	Time stamps of segments
RF01	47	14:14:13-14:14:13; 14:14:27-14:14:44; 14:19:09-14:19:27; 14:20:23-14:20:42; 14:29:22-14:29:34; 14:31:57-14:32:07; 15:33:52-15:34:02; 15:37:06-15:37:15; 15:41:55-15:42:08; 15:47:13-15:47:22; 15:48:04-15:48:21; 15:51:19-15:51:28; 15:54:24-15:54:35; 15:56:04-15:56:13; 15:59:58-16:00:21; 16:05:47-16:06:07; 16:06:17-16:06:34; 16:14:22-16:14:45; 16:27:38-16:27:47; 16:29:25-16:29:39; 16:29:59-16:30:20; 16:31:28-16:31:43; 16:35:07-16:35:16; 16:35:30-16:35:39; 16:36:13-16:36:26; 16:37:29-16:37:39; 16:38:42-16:39:24; 16:41:13-16:41:28; 16:50:18-16:50:28; 16:56:29-16:56:41; 16:57:10-16:57:25; 17:03:44-17:04:12; 17:04:39-17:04:50; 17:05:06-17:05:17; 17:06:42-17:06:56; 17:11:44-17:11:57; 17:12:16-17:12:29; 17:13:42-17:13:56; 17:14:03-17:14:22; 17:18:57-17:19:09; 17:19:57-17:20:14; 17:43:17-17:43:27; 17:43:51-17:44:00; 17:49:39-17:49:48; 17:51:47-17:52:02; 18:22:24-18:22:36; 18:23:38-18:23:48
RF02	38	15:49:19-15:49:19; 15:49:51-15:50:01; 15:51:22-15:51:40; 15:56:08-15:56:26; 15:58:06-15:58:16; 15:58:18-15:58:32; 16:01:10-16:01:29; 16:01:41-16:01:55; 16:05:20-16:05:36; 16:07:30-16:07:39; 16:10:17-16:10:46; 16:12:58-16:13:12; 16:19:13-16:19:23; 16:25:01-16:25:15; 16:25:32-16:26:00; 16:43:13-16:44:15; 17:08:16-17:08:30; 17:09:13-17:09:26; 17:37:38-17:37:57; 17:38:20-17:38:29; 17:51:30-17:51:45; 17:51:49-17:52:01; 17:53:37-17:53:48; 18:46:44-18:46:54; 18:47:20-18:47:37; 18:51:45-18:51:57; 18:53:18-18:53:32; 18:54:07-18:54:21; 18:56:30-18:56:41; 18:56:44-18:56:59; 18:58:43-18:58:55; 18:59:33-18:59:43; 19:01:17-19:01:46; 19:01:59-19:02:20; 19:06:02-19:06:17; 19:08:00-19:08:33; 19:08:49-19:09:30; 19:10:06-19:10:25
RF03	39	23:01:28-23:01:28; 23:02:53-23:03:03; 23:07:10-23:07:22; 23:34:03-23:34:24; 23:34:33-23:34:43; 23:36:01-23:36:15; 23:37:44-23:37:53; 23:40:38-23:40:50; 23:41:42-23:41:54; 23:46:12-23:46:25; 23:49:27-23:49:41; 23:52:45-23:53:10; 23:54:51-23:55:12; 23:56:39-23:56:52; 23:57:51-23:58:05; 23:59:42-23:59:58; 00:00:02-00:00:13; 00:00:43-00:00:56; 00:00:59-00:01:08; 00:03:01-00:03:10; 00:04:41-00:04:50; 00:04:55-00:05:20; 00:07:42-00:07:51; 00:09:29-00:09:43; 00:12:20-00:12:40; 00:14:47-00:15:33; 00:44:17-00:44:53; 00:45:16-00:45:33; 00:48:21-00:48:47; 00:48:53-00:49:04; 00:49:06-00:49:19; 00:51:03-00:51:31; 00:51:36-00:51:45; 00:56:33-00:56:43; 00:59:19-00:59:28; 00:59:36-00:59:51; 01:00:05-01:00:24; 01:01:03-01:01:44; 01:04:10-01:04:49
RF04	33	20:07:43-20:07:43; 20:08:45-20:08:59; 20:16:33-20:16:46; 20:17:02-20:17:13; 20:19:05-20:19:14; 20:19:21-20:19:37; 20:23:03-20:23:16; 20:25:56-20:26:05; 20:27:25-20:27:35; 20:28:11-20:28:22; 20:29:34-20:29:56; 20:30:10-20:30:35; 20:31:14-20:31:25; 20:32:00-20:32:15; 20:33:40-20:34:40; 20:50:24-20:50:43; 21:39:01-21:39:15; 21:40:30-21:40:50; 21:42:56-21:43:29; 21:44:42-21:45:01; 21:45:30-21:45:52; 21:46:48-21:47:09; 21:48:31-21:48:47; 21:50:14-21:50:32; 21:53:56-21:54:09; 21:56:00-21:56:09; 21:57:17-21:57:27; 22:05:07-22:05:23; 22:06:54-22:07:24; 22:17:45-22:17:56; 22:19:51-22:20:20; 22:21:47-22:21:59; 22:22:15-22:22:36
RF05	28	15:38:32-15:38:32; 15:38:55-15:39:05; 15:39:20-15:39:29; 15:42:27-15:42:40; 15:42:53-15:43:03; 15:45:10-15:45:24; 15:45:33-15:45:43; 15:46:17-15:46:37; 15:50:12-15:50:25; 15:51:14-15:51:33; 16:57:27-16:57:38; 16:58:03-16:58:14; 17:06:10-17:06:24; 17:08:14-17:08:25; 17:13:45-17:13:54; 17:14:46-17:15:00; 17:20:05-17:20:16; 17:25:11-17:25:31; 17:26:11-17:26:26; 17:29:54-17:30:03; 17:34:37-17:34:58; 17:36:01-17:36:10; 17:36:48-17:36:59; 18:08:43-18:08:54; 18:14:45-18:14:58; 18:21:02-18:21:15; 18:26:49-18:27:02; 18:28:29-18:28:38
RF06	11	21:17:48-21:17:48; 21:19:51-21:20:24; 21:22:11-21:22:28; 21:23:49-21:24:06; 21:24:51-21:25:18; 22:07:54-22:08:12; 22:09:53-22:10:15; 22:11:35-22:11:50; 22:13:37-22:13:49; 22:14:31-22:14:48; 22:16:21-22:16:31

Table S1: Table of times for the flight segments analyzed in the main paper for flights RF01-RF06.

These segments are automatically selected using the liquid water content and length thresholds for warm clouds.

Flight no.	No. of seg.	Time stamps of segments
RF07	35	21:09:58-21:09:58; 21:16:58-21:17:16; 21:17:29-21:17:53; 21:19:34-21:19:46; 21:33:33-21:33:58; 21:36:37-21:36:47; 21:38:36-21:38:46; 21:39:09-21:39:29; 21:39:38-21:39:47; 21:39:59-21:40:26; 21:41:12-21:41:22; 21:42:31-21:42:41; 21:44:55-21:45:23; 21:49:18-21:49:31; 22:14:51-22:15:03; 22:17:34-22:17:45; 23:01:05-23:01:54; 23:02:58-23:03:17; 23:03:23-23:03:34; 23:03:39-23:03:48; 23:06:14-23:06:46; 23:08:33-23:08:49; 23:11:05-23:11:17; 23:11:34-23:11:48; 23:11:54-23:12:03; 23:12:10-23:13:02; 23:14:52-23:15:04; 23:15:55-23:16:11; 23:16:31-23:16:41; 23:16:43-23:17:03; 23:21:02-23:21:12; 23:23:00-23:23:12; 23:23:14-23:23:25; 23:25:29-23:26:05; 23:26:15-23:26:35
RF08	48	21:15:58-21:15:58; 21:16:28-21:16:38; 21:18:06-21:18:42; 21:21:42-21:21:56; 21:23:57-21:24:13; 21:24:15-21:24:43; 21:29:44-21:29:54; 21:29:56-21:30:50; 21:30:52-21:31:07; 21:33:38-21:33:52; 21:34:40-21:34:56; 21:38:01-21:38:11; 21:38:51-21:39:22; 21:39:45-21:40:03; 21:41:06-21:41:26; 21:42:51-21:43:03; 21:43:18-21:43:33; 21:47:16-21:47:26; 21:47:28-21:47:42; 21:48:22-21:48:58; 21:50:10-21:50:34; 21:51:38-21:51:47; 22:00:03-22:00:14; 22:00:31-22:00:44; 22:01:25-22:01:35; 22:01:54-22:02:08; 22:02:14-22:02:26; 22:02:28-22:02:40; 22:03:41-22:03:55; 22:05:32-22:05:56; 22:06:29-22:06:38; 22:06:45-22:06:59; 22:07:48-22:08:06; 22:08:10-22:08:25; 22:09:33-22:09:50; 22:10:18-22:10:54; 22:13:37-22:14:10; 22:15:43-22:16:02; 22:16:48-22:16:58; 22:17:13-22:18:11; 22:18:13-22:18:28; 22:19:21-22:20:06; 22:20:23-22:21:24; 22:21:59-22:22:38; 22:22:53-22:23:10; 22:23:12-22:23:36; 22:23:48-22:24:05; 22:35:46-22:36:26
RF09	1	21:45:45-21:45:45
RF11	42	17:00:27-17:00:27; 17:02:13-17:02:24; 17:13:23-17:13:48; 17:29:09-17:29:18; 17:30:50-17:31:03; 17:38:48-17:38:58; 17:41:41-17:41:50; 17:43:36-17:43:54; 17:46:30-17:46:49; 17:47:35-17:47:49; 18:04:12-18:04:38; 18:05:28-18:05:46; 18:06:14-18:06:24; 18:06:28-18:06:51; 18:07:57-18:08:09; 18:08:12-18:08:44; 18:08:47-18:09:34; 18:11:00-18:11:09; 18:11:31-18:12:03; 18:12:08-18:12:18; 18:13:08-18:13:19; 18:13:21-18:13:30; 18:13:32-18:13:51; 18:14:59-18:15:22; 18:16:04-18:16:13; 18:16:48-18:17:08; 18:17:43-18:17:54; 18:18:46-18:18:55; 18:20:59-18:21:08; 18:22:02-18:22:13; 18:22:26-18:22:49; 18:29:28-18:29:50; 18:30:46-18:30:58; 18:33:44-18:34:01; 18:34:28-18:34:45; 18:34:53-18:35:03; 18:39:46-18:39:56; 18:42:05-18:42:18; 18:52:46-18:53:03; 18:55:12-18:55:33; 18:56:23-18:56:38; 18:58:31-18:59:05
RF12	30	21:33:06-21:33:06; 21:34:18-21:34:39; 21:36:56-21:37:44; 21:39:59-21:40:15; 21:40:17-21:40:28; 21:41:55-21:42:08; 21:42:10-21:42:22; 21:42:33-21:42:48; 21:45:13-21:45:39; 21:49:45-21:50:04; 21:50:21-21:50:34; 21:56:12-21:56:22; 21:57:48-21:58:17; 22:49:25-22:50:01; 22:52:43-22:54:05; 23:32:44-23:33:46; 23:33:53-23:34:09; 23:34:36-23:34:52; 23:34:54-23:35:15; 23:42:07-23:42:34; 23:43:16-23:43:44; 23:44:05-23:44:38; 23:46:27-23:46:42; 00:05:10-00:06:13; 00:11:37-00:12:13; 00:14:10-00:14:35; 00:16:59-00:17:15; 00:20:09-00:20:24; 00:21:47-00:22:03; 00:22:13-00:22:29
RF13	43	17:35:55-17:35:55; 17:37:09-17:37:19; 17:45:06-17:45:18; 17:46:03-17:46:22; 17:47:01-17:47:13; 17:50:00-17:50:23; 17:51:20-17:51:35; 17:52:27-17:52:38; 17:52:58-17:53:09; 17:53:42-17:53:53; 17:54:00-17:54:21; 17:54:27-17:55:01; 17:55:22-17:55:37; 17:57:14-17:57:42; 17:58:06-17:58:19; 17:59:57-18:00:06; 18:00:12-18:00:25; 18:01:45-18:01:58; 18:03:19-18:03:41; 18:29:47-18:30:19; 18:32:17-18:32:36; 18:35:56-18:36:06; 18:41:05-18:41:14; 18:42:20-18:42:30; 18:43:05-18:43:34; 18:43:56-18:44:05; 18:44:25-18:44:44; 18:44:49-18:45:00; 18:48:09-18:48:49; 18:49:20-18:49:41; 18:49:43-18:50:16; 18:51:01-18:51:10; 18:51:32-18:51:48; 18:52:48-18:53:11; 18:53:54-18:54:27; 18:55:03-18:55:17; 18:55:39-18:55:54; 18:56:12-18:56:42; 18:58:15-18:58:27; 18:58:49-18:58:58; 18:59:02-18:59:17; 19:00:09-19:00:31; 19:01:37-19:01:47

Table S2: Same as Table S1 but for flights RF07-RF13

Input	Value	Unit
Domain length, Lx	1000	m
Grid size, dx	10	m
Gamma dist mean diameter, $\bar{d}$	15	μm
Shape parameter, k	25	n.d.
No. of cloud droplets in the sample	1000	n.d.
Cloud drop number concentration, n_d	200	cm^{-3}
RH_{env0}	0.5	n.d.
Water vapor mixing ratio, q_{v0}	10	g kg^{-1}
Pressure, P	563	hPa
Imposed temperature difference between cloud and env(K), ΔT	1	K
Cloud mixing fraction, χ	0.5	n.d.
Eddy dissipation rate, ε	0.008	$m^2 s^{-3}$

Table S3: Input values to the turbulent-diffusion-evaporation model

REFERENCES

1. J. S. Turner, *Buoyancy Effects in Fluids* (Cambridge Univ. Press, 2012).

2. R. A. Houze Jr, *Cloud Dynamics* (Academic Press, 1993).

3. N. Maury, G. C. Roberts, F. Couvreux, T. Verdu, P. Narvor, S. Lacroix, G. Hattenberger, Quantifying the mixing of trade-wind cumulus during the NEPHELAE-EUREC4A field campaign with remotely piloted aircraft. *Q. J. Roy. Meteorol. Soc.* **149**, 809–829 (2023).

4. E. Eytan, Y. Arieli, A. Khain, O. Altaratz, M. Pinsky, E. Gavze, I. Koren, The role of the toroidal vortex in cumulus clouds' entrainment and mixing. *J. Geophys. Res. Atmos.* **129**, e2023JD039493 (2024).

5. W. C. de Rooy, P. Bechtold, K. Fröhlich, C. Hohenegger, H. Jonker, D. Mironov, A. Pier Siebesma, J. Teixeira, J.-I. Yano, Entrainment and detrainment in cumulus convection: An overview. *Q. J. Roy. Meteorol. Soc.* **139**, 1–19 (2013).

6. S. Bera, G. Pandithurai, T. V. Prabha, Entrainment and droplet spectral characteristics in convective clouds during transition to monsoon. *Atmos. Sci. Lett.* **17**, 286–293 (2016).

7. C. Lu, Y. Liu, X. Xu, S. Gao, C. Sun, *Entrainment, Mixing, and Their Microphysical Influences* (American Geophysical Union, 2023), chap. 4, pp. 87–120; https://doi.org/10.1002/9781119529019.ch4.

8. F. Hoffmann, *The Small-Scale Mixing of Clouds With Their Environment: Impacts on Micro- and Macroscale Cloud Properties* (American Geophysical Union, 2023), chap. 12, pp. 255–270; https://doi.org/10.1002/9781119700357.ch12.

9. J.-S. Lim, F. Hoffmann, Between broadening and narrowing: How mixing affects the width of the droplet size distribution. *J. Geophys. Res. Atmos.* **128**, e2022JD037900 (2023).

10. J. Latham, R. L. Reed, Laboratory studies of the effects of mixing on the evolution of cloud droplet spectra. *Q. J. Roy. Meteorol. Soc.* **103**, 297–306 (1977).

11. M. B. Baker, R. G. Corbin, J. Latham, The influence of entrainment on the evolution of cloud droplet spectra: I. A model of inhomogeneous mixing. *Q. J. Roy. Meteorol. Soc.* **106**, 581–598 (1980).

12. A. Arakawa, W. H. Schubert, Interaction of a cumulus cloud ensemble with the large-scale environment, part I. *J. Atmos. Sci.* **31**, 674–701 (1974).

13. M. Tiedtke, A comprehensive mass flux scheme for cumulus parameterization in large-scale models. *Mon. Weather Rev.* **117**, 1779–1800 (1989).

14. X. Xu, C. Lu, Y. Liu, S. Luo, X. Zhou, S. Endo, L. Zhu, Y. Wang, Influences of an entrainment–mixing parameterization on numerical simulations of cumulus and stratocumulus clouds. *Atmos. Chem. Phys.* **22**, 5459–5475 (2022).

15. C. Lu, Y. Liu, S. Niu, S. Krueger, T. Wagner, Exploring parameterization for turbulent entrainment-mixing processes in clouds. *J. Geophys. Res. Atmos.* **118**, 185–194 (2013).

16. D. Jarecka, W. W. Grabowski, H. Morrison, H. Pawlowska, Homogeneity of the subgrid-scale turbulent mixing in large-eddy simulation of shallow convection. *J. Atmos. Sci.* **70**, 2751–2767 (2013).

17. D. Jarecka, W. W. Grabowski, H. Pawlowska, Modeling of subgrid-scale mixing in large-eddy simulation of shallow convection. *J. Atmos. Sci.* **66**, 2125–2133 (2009).

18. X. He, C. Lu, Y. Liu, Y. Zhu, Y. Peng, L. Zhu, X. Xu, S. Luo, H. Wang, T. Li, J. Li, H. Wang, S. Gao, Y. Lin, Implementation and evaluation of physics-driven dynamic entrainment-mixing parameterization in a climate model and its impact on low-cloud simulation. *J. Geophys. Res. Atmos.* **130**, e2024JD041918 (2025).

19. J.-S. Lim, F. Hoffmann, Life cycle evolution of mixing in shallow cumulus clouds. *J. Geophys. Res. Atmos.* **129**, e2023JD040393 (2024).

20. K. J. Sanchez, G. C. Roberts, R. Calmer, K. Nicoll, E. Hashimshoni, D. Rosenfeld, J. Ovadnevaite, J. Preissler, D. Ceburnis, C. O'Dowd, L. M. Russell, Top-down and bottom-up

aerosol–cloud closure: Towards understanding sources of uncertainty in deriving cloud shortwave radiative flux. *Atmos. Chem. Phys.* **17**, 9797–9814 (2017).

21. K. J. Sanchez, G. C. Roberts, M. Diao, L. M. Russell, Measured constraints on cloud top entrainment to reduce uncertainty of nonprecipitating stratocumulus shortwave radiative forcing in the Southern Ocean. *Geophys. Res. Lett.* **47**, e2020GL090513 (2020).

22. K. Lehmann, H. Siebert, R. A. Shaw, Homogeneous and inhomogeneous mixing in cumulus clouds: Dependence on local turbulence structure. *J. Atmos. Sci.* **66**, 3641–3659 (2009).

23. C. Lu, Y. Liu, S. Niu, S. Endo, Scale dependence of entrainment-mixing mechanisms in cumulus clouds. *J. Geophys. Res. Atmos.* **119**, 13877–13890 (2014).

24. S. S. Yum, J. Wang, Y. Liu, G. Senum, S. Springston, R. McGraw, J. M. Yeom, Cloud microphysical relationships and their implication on entrainment and mixing mechanism for the stratocumulus clouds measured during the VOCALS project. *J. Geophys. Res. Atmos.* **120**, 5047–5069 (2015).

25. M. A. Beals, J. P. Fugal, R. A. Shaw, J. Lu, S. M. Spuler, J. L. Stith, Holographic measurements of inhomogeneous cloud mixing at the centimeter scale. *Science* **350**, 87–90 (2015).

26. J. M. Yeom, S. S. Yum, Y. Liu, C. Lu, A study on the entrainment and mixing process in the continental stratocumulus clouds measured during the RACORO campaign. *Atmos. Res.* **194**, 89–99 (2017).

27. B. Kumar, P. Götzfried, N. Suresh, J. Schumacher, R. A. Shaw, Scale dependence of cloud microphysical response to turbulent entrainment and mixing. *J. Adv. Model. Earth Syst.* **10**, 2777–2785 (2018).

28. J. M. Yeom, S. S. Yum, R. A. Shaw, I. la, J. Wang, C. Lu, Y. Liu, F. Mei, B. Schmid, A. Matthews, Vertical variations of cloud microphysical relationships in marine stratocumulus clouds observed during the ACE-ENA campaign. *J. Geophys. Res. Atmos.* **126**, e2021JD034700 (2021).

29. K. Ranjbar, K. Bala, M. Wolde, ESCAPE: CDP and FCDP Scattering Probe Data version 1.0 (NSF, 2023); https://data.eol.ucar.edu/dataset/619.014.

30. U.-E. O. Laboratory, Holographic Detector for Clouds version 1.0 (NSF, 2022); https://data.eol.ucar.edu/dataset/619.002.

31. P. Kollias, G. M. Mc Farquhar, E. Bruning, P. J. De Mott, M. R. Kumjian, P. Lawson, Z. Lebo, T. Logan, K. Lombardo, M. Oue, G. Roberts, R. A. Shaw, S. C. van den Heever, M. Wolde, K. R. Barry, D. Bodine, R. Bruintjes, V. Chandrasekar, A. Dzambo, T. C. J. Hill, M. Jensen, F. Junyent, S. M. Kreidenweis, K. Lamer, E. Luke, A. Bansemer, C. M. Cluskey, L. Nichman, C. Nguyen, R. J. Patnaude, R. J. Perkins, H. Powers, K. Ranjbar, E. Roux, J. Snyder, B. P. Treserras, P. Tsai, N. A. Wales, C. Wolf, N. Allwayin, B. Ascher, J. Barr, Y. Hu, Y. Huang, M. Litzmann, Z. Mages, K. M. Keown, S. Patil, E. Rosky, K. Tuftedal, M.-D. Tzeng, Z. Zhu, Experiment of Sea Breeze Convection, Aerosols, Precipitation and Environment (ESCAPE). *Bull. Am. Meteorol. Soc.* **106**, E310–E332 (2024).

32. S. Lance, C. A. Brock, D. Rogers, J. A. Gordon, Water droplet calibration of the cloud droplet probe (CDP) and in-flight performance in liquid, ice and mixed-phase clouds during ARCPAC. *Atmos. Meas. Tech.* **3**, 1683–1706 (2010).

33. S. M. Spuler, J. Fugal, Design of an in-line, digital holographic imaging system for airborne measurement of clouds. *Appl. Optics* **50**, 1405–1412 (2011).

34. J. B. Jensen, P. H. Austin, M. B. Baker, A. M. Blyth, Turbulent mixing, spectral evolution and dynamics in a warm cumulus cloud. *J. Atmos. Sci.* **42**, 173–192 (1985).

35. F. Burnet, J.-L. Brenguier, Observational study of the entrainment-mixing process in warm convective clouds. *J. Atmos. Sci.* **64**, 1995–2011 (2007).

36. A. Korolev, A. Khain, M. Pinsky, J. French, Theoretical study of mixing in liquid clouds – Part 1: Classical concepts. *Atmos. Chem. Phys.* **16**, 9235–9254 (2016).

37. M. Pinsky, A. Khain, Theoretical analysis of mixing in liquid clouds—Part IV: DSD evolution and mixing diagrams. *Atmos. Chem. Phys.* **18**, 3659–3676 (2018).

38. J. Fries, G. Sardina, G. Svensson, B. Mehlig, Key parameters for droplet evaporation and mixing at the cloud edge. *Q. J. Roy. Meteorol. Soc.* **147**, 2160–2172 (2021).

39. M. Pinsky, A. Khain, A. Korolev, Theoretical analysis of mixing in liquid clouds—Part 3: Inhomogeneous mixing. *Atmos. Chem. Phys.* **16**, 9273–9297 (2016).

40. H. E. Gerber, G. M. Frick, J. B. Jensen, J. G. Hudson, Entrainment, mixing, and microphysics in trade-wind cumulus. *J. Meteorol. Soc. Jpn.* **86A**, 87–106 (2008).

41. C. Siewert, J. Bec, G. Krstulovic, Statistical steady state in turbulent droplet condensation. *J. Fluid Mech.* **810**, 254–280 (2017).

42. J. C. Wyngaard, *Turbulence in the Atmosphere* (Cambridge Univ. Press, 2011).

43. N. Allwayin, M. L. Larsen, S. Glienke, R. A. Shaw, Locally narrow droplet size distributions are ubiquitous in stratocumulus clouds. *Science* **384**, 528–532 (2024).

44. J. M. Yeom, I. Helman, P. Prabhakaran, J. C. Anderson, F. Yang, R. A. Shaw, W. Cantrell, Cloud microphysical response to entrainment and mixing is locally inhomogeneous and globally homogeneous: Evidence from the lab. *Proc. Natl. Acad. Sci. U.S.A.* **120**, e2307354120 (2023).

45. C.-W. Su, S. K. Krueger, P. A. McMurtry, P. H. Austin, Linear eddy modeling of droplet spectral evolution during entrainment and mixing in cumulus clouds. *Atmos. Res.* **47**, 41–58 (1998).

46. J. Katzwinkel, H. Siebert, T. Heus, R. A. Shaw, Measurements of turbulent mixing and subsiding shells in trade wind cumuli. *J. Atmos. Sci.* **71**, 2810–2822 (2014).

47. E. Freud, D. Rosenfeld, J. R. Kulkarni, Resolving both entrainment-mixing and number of activated CCN in deep convective clouds. *Atmos. Chem. Phys.* **11**, 12887–12900 (2011).

48. R. Calmer, G. C. Roberts, K. J. Sanchez, J. Sciare, K. Sellegri, D. Picard, M. Vrekoussis, M. Pikridas, Aerosol–cloud closure study on cloud optical properties using remotely piloted aircraft

measurements during a BACCHUS field campaign in Cyprus. *Atmos. Chem. Phys.* **19**, 13989–14007 (2019).

49. H. Gerber, G. Frick, S. P. Malinowski, H. Jonsson, D. Khelif, S. K. Krueger, Entrainment rates and microphysics in POST stratocumulus. *J. Geophys. Res. Atmos.* **118**, 12094–12109 (2013).

50. C. Lu, Y. Liu, S. Niu, Examination of turbulent entrainment-mixing mechanisms using a combined approach. *J. Geophys. Res. Atmos.* **116**, 10.1029/2011JD015944 (2011).

51. G. C. Roberts, M. V. Ramana, C. Corrigan, D. Kim, V. Ramanathan, Simultaneous observations of aerosol-cloud-albedo interactions with three stacked unmanned aerial vehicles. *Proc. Natl. Acad. Sci. U.S.A.* **105**, 7370–7375 (2008).

52. H. Pawlowska, J. Brenguier, F. Burnet, Microphysical properties of stratocumulus clouds. *Atmos. Res.* **55**, 15–33 (2000).

53. N. Desai, Y. Liu, S. Glienke, R. A. Shaw, C. Lu, J. Wang, S. Gao, Vertical variation of turbulent entrainment mixing processes in marine stratocumulus clouds using high-resolution digital holography. *J. Geophys. Res. Atmos.* **126**, e2020JD033527 (2021).

54. J. D. Small, P. Y. Chuang, H. H. Jonsson, Microphysical imprint of entrainment in warm cumulus. *Tellus B, Chem. Phys. Meteorol.* **65**, 19922 (2022).

55. C. A. Jeffery, Inhomogeneous cloud evaporation, invariance, and Damköhler number. *J. Geophys. Res.* **112**, 10.1029/2007JD008789 (2007).

56. D. P. Grosvenor, O. Sourdeval, P. Zuidema, A. Ackerman, M. D. Alexandrov, R. Bennartz, R. Boers, B. Cairns, J. C. Chiu, M. Christensen, H. Deneke, M. Diamond, G. Feingold, A. Fridlind, A. Hünerbein, C. Knist, P. Kollias, A. Marshak, D. McCoy, D. Merk, D. Painemal, J. Rausch, D. Rosenfeld, H. Russchenberg, P. Seifert, K. Sinclair, P. Stier, B. van Diedenhoven, M. Wendisch, F. Werner, R. Wood, Z. Zhang, J. Quaas, Remote sensing of droplet number concentration in warm clouds: A review of the current state of knowledge and perspectives. *Rev. Geophys.* **56**, 409–453 (2018).

57. K. Bala, M. Wolde, ESCAPE: Atmospheric and Aircraft State Data version 1.0 (NSF, 2023); https://data.eol.ucar.edu/dataset/619.015.

58. F. Yang, R. Shaw, H. Xue, Conditions for super-adiabatic droplet growth after entrainment mixing. *Atmos. Chem. Phys.* **16**, 9421–9433 (2016).

59. R. A. Shaw, K. K. Chandrakar, P. Prabhakaran, H. Siebert, F. Yang, Turbulence in clouds, in *Environmental Turbulence*, E. Bou-Zeid, S. Sarkar, Eds. (Academic Press, 2025), chap. 14; https://shop.elsevier.com/books/environmental-turbulence/bou-zeid/978-0-323-95873-8.
